# Identifying specific TLS-associated genes as potential biomarkers for predicting prognosis and evaluating the efficacy of immunotherapy in soft tissue sarcoma

**DOI:** 10.3389/fimmu.2024.1372692

**Published:** 2024-04-24

**Authors:** Xiang-Xu Wang, Yun-Peng Liu, Yajie Lu, Li-Hong Wu, Jing-Yi Ren, Hongchen Ji, Xiaowen Wang, Hong-Mei Zhang

**Affiliations:** ^1^ Department of Clinical Oncology, Xijing Hospital, Fourth Military Medical University, Xi’an, Shaanxi, China; ^2^ Xijing 986 Hospital Department, Fourth Military Medical University, Xi’an, Shaanxi, China

**Keywords:** soft tissue sarcoma, tertiary lymphatic structure, single-cell sequencing analysis, prognosis signature, immunotherapy

## Abstract

**Background:**

The tertiary lymphatic structure (TLS) is an important component of the tumor immune microenvironment and has important significance in patient prognosis and response to immune therapy. However, the underlying mechanism of TLS in soft tissue sarcoma remains unclear.

**Methods:**

A total of 256 RNAseq and 7 single-cell sequencing samples were collected from TCGA-SARC and GSE212527 cohorts. Based on published TLS-related gene sets, four TLS scores were established by GSVA algorithm. The immune cell infiltration was calculated via TIMER2.0 and “MCPcounter” algorithms. In addition, the univariate, LASSO, and multivariate-Cox analyses were used to select TLS-related and prognosis-significant hub genes. Single-cell sequencing dataset, clinical immunohistochemical, and cell experiments were utilized to validate the hub genes.

**Results:**

In this study, four TLS-related scores were identified, and the total-gene TLS score more accurately reflected the infiltration level of TLS in STS. We further established two hub genes (DUSP9 and TNFSF14) prognosis markers and risk scores associated with soft tissue sarcoma prognosis and immune therapy response. Flow cytometry analysis showed that the amount of CD3, CD8, CD19, and CD11c positive immune cell infiltration in the tumor tissue dedifferentiated liposarcoma patients was significantly higher than that of liposarcoma patients. Cytological experiments showed that soft tissue sarcoma cell lines overexpressing TNFSF14 could inhibit the proliferation and migration of sarcoma cells.

**Conclusion:**

This study systematically explored the TLS and related genes from the perspectives of bioinformatics, clinical features and cytology experiments. The total-gene TLS score, risk score and TNFSF14 hub gene may be useful biomarkers for predicting the prognosis and immunotherapy efficacy of soft tissue sarcoma.

## Introduction

Soft tissue sarcoma (STS) is a highly heterogeneous malignant tumor originating from mesenchymal tissue, accounting for approximately 0.72% to 1.05% of human malignant tumors (1). The treatment of early-stage STS primarily relies on surgery, with a 5-year overall survival rate ranging from 60% to 80% (2). However, more than half of the patients still experience local recurrence or distant metastasis after surgery, and the average survival period for patients with distant metastasis is only 12 to 18 months (2). Treatment options for advanced STS are limited, and the standard first-line therapy is chemotherapy based on anthracyclines, but the response rate is only 10% to 25% (3). Molecularly targeted drugs, such as Anlotinib and Pazopanib, are second-line treatments for advanced STS, but the median progression-free survival is only 5.6 months (4, 5).

Immunotherapy, a departure from traditional treatment methods that directly eliminate tumor cells, relies on stimulating the host’s immune system to harness its natural defenses against malignant tumor cells (6). Immunotherapy has achieved positive results in various cancers and even changed the treatment direction and outcome of refractory tumors such as malignant melanoma (7). Currently, STS immunotherapy has achieved certain efficacy in some histological types, but the overall situation remains concerning (8). There is an urgent need to further explore the STS tumor immune microenvironment and identify more effective immunotherapy strategies and prognostic biomarkers (9, 10).

TLS, also known as ectopic lymphoid organs, are large lymphatic tissues that exhibit architectural similarities to lymphoid nodes. TLS develops in non-lymphatic tissues characterized by persistent inflammation, such as chronic infections, organ transplantation, autoimmune diseases, and cancer sites (11, 12). TLS has a germinal center composed of B cells, follicular dendritic cells, and high endothelial small veins, and these structural characteristics are closely related to adaptive immune response (13). TLS serves as critical sites for the continuous production of effector immune cells, thereby playing a crucial role in shaping the immune microenvironment of tumors. TLS contributes to the promotion of T and B cells, which are essential for mounting an effective immune response against tumors (14).

TLS also plays a vital role in soft tissue sarcoma, but the specific mechanisms remain elusive. In this study, we screened three sets of TLS-related genes based on previous literature and combined them into a comprehensive total gene set. Utilizing the GSVA algorithm, we assessed the abundance of tertiary lymphoid structures in STS. An STS-related prognostic score was established, and two hub genes were identified. These results were corroborated using single-cell and spatial transcriptome data, as well as patient tissues. This study enhances our understanding of tertiary lymphoid structures and provides a potential mechanistic basis for immunotherapy in soft tissue sarcoma.

## Materials and methods

### Data collection and preprocessing

A total of 256 patients with STS tumors were collected from The Cancer Genome Atlas (TCGA) database, and the detailed clinical information is listed in **Table 1**. The single cell transcriptome data of 3 undifferentiated pleomorphic sarcoma (UPS) and 4 leiomyosarcomas (LMS) samples were collected from the GSE212527 cohort in Gene Expression Omnibus (GEO) database (https://www.ncbi.nlm.nih.gov/). We performed “harmony” algorithm to remove batch effects among different Single cell samples, and further used “Principal Component Analysis, PCA” dimensionality reduction clustering to obtain cell subpopulations, and then applied “singleR” algorithm to annotate cell names. In addition, two STS patients under immunotherapy, were used for immunohistochemical of two hub genes, DUSP9 and TNFSF14. This study was approved were approved by Ethics Committee of the First Affiliated Hospital of Air Force Medical University (ethics number: XJLL-KY2184).

**Table 1:** Clinical features between high and low TLS subtypes in the SARC cohort.

Variables n(%)	Overall	TLS_H	TLS_L	*P*
	256(100)	128(50)	128(50)	
**Age (years)**				**0.001**
≤50	51 (19.9)	14 (10.9)	37 (28.9)	
51-70	142 (55.5)	75 (58.6)	67 (52.3)	
>70	63 (24.6)	39 (30.5)	24 (18.8)	
**Gender**				**<0.001**
Male	117 (45.7)	74 (57.8)	43 (33.6)	
Female	139 (54.3)	54 (42.2)	85(66.4)	
**Race**				0.571
White	224 (87.5)	114 (89.1)	110 (85.9)	
Other	32 (12.5)	14 (10.9)	18 (14.1)	
**Histological**				**<0.001**
DDL	58 (22.7)	39 (30.5)	19 (14.8)	
LMS	103 (40.2)	40 (31.2)	63 (49.2)	
MFS	25 (9.8)	18 (14.1)	7 (5.5)	
UPS	49 (19.1)	28 (21.9)	21 (16.4)	
Other	21 (8.2)	3 (2.3)	18 (14.1)	
**Vital status**				**0.029**
Dead	98 (38.3)	40 (31.2)	58 (45.3)	
Alive	158 (61.7)	88 (68.8)	70 (54.7)	
**Tumor status**				0.172
Tumor free	125 (48.8)	70 (54.7)	55 (43.0)	
With tumor	122 (47.7)	54 (42.2)	68 (53.1)	
NA	9 (3.5)	4 (3.1)	5 (3.9)	
**Surgical margin**				0.237
R0	153 (59.8)	79 (61.7)	74 (57.8)	
R1	67 (26.2)	36 (28.1)	31 (24.2)	
R2	9 (3.5)	2 (1.6)	7 (5.5)	
RX	27 (10.5)	11 (8.6)	16 (12.5)	

DDL, Dedifferentiated liposarcoma; LMS, leiomyosarcomas; MFS, Myxofibrosarcoma; UPS, undifferentiated pleomorphic sarcoma; NA, not available.The P value in bold indicated that "P<0.05".

### Four TLS signatures estimation

Four TLS-related gene sets collected from the previous studies, 12 Cytokines genes (15), 9gene set (CD79B, CD1D, CCR6, LAT, SKAP1, CETP, EIF1AY, RBP5, and PTGDS) (16), hallmark genes (CCL19, CCL21, CXCL13, CCR7, CXCR5, SELL, and LAMP3) (17) and the total above 28 genes after removing duplicates. In this study, the R package “GSVA” (18) was used to estimate the variation of four TLS-related gene sets and obtained the score for each TCGA sample, which was defined as the TLS score. In this study, we utilized the R package “GSVA” (18) to assess the variability of four TLS-related gene sets. We computed the TLS score for each TCGA sample serves as a quantification of the TLS-related characteristics in the tumor immune microenvironment.

### Immune microenvironment assessment and immune subtype identification

We performed the “ESTIMATE” R package and calculated the immune, stromal score, and tumor purity of each sample (19). We investigated the relationship of TLS score with tumor purity and immune cell infiltration in each sample by TIMER 2.0 (20) (http://timer.cistrome.org/) and CIBERSORT algorithms (21). The patients in the TCGA-SARC cohort were identified into 5 subtypes (C1: wound healing, C2: IFN-γ-dominant, C3: inflammatory, C4: lymphocyte-depleted, and C6: TGF-β-dominant subtypes) of tumors based on previous studies (22). And the C5 subtype (immunologically quiet subtype) was not identified in the TCGA-SARC cohort.

### Identification of differentially expressed genes (DEGs) and enrichment analysis

We defined two TLS subgroups (TLS-H and TLS-L) by the median of the total TLS genes score. The DEGs between TLS-H and TLS-L subgroups in STS samples with FDR < 0.05 and |log FC| > 2 were chosen for further analysis. We performed Kyoto Encyclopedia of Genes and Genomes (KEGG) via the “ClusterProfiler” package, and conducted pathway enrichment analysis to identify the function of DEGs.

### Construction and validation of the prognostic TLS-related genes risk score

The prognostic TLS-related genes (*P*<0.05) were selected by univariate Cox regression analysis based on selected DEGs in the TCGA training cohort. Two prognostic TLS-related hub genes (DUSP9 and TNFSF14) were finally screened for LASSO and multivariate COX regression analysis. The prognosis risk score for each STS patient was calculated by the formula: Risk score = 0.319*Exp (DUSP9)-1.28*Exp (TNFSF14).

### Immunohistochemistry of DUSP9 and TNFSF14

The antibodies DUSP9 and TNFSF14 used were purchased from Proteintech company, and the staining steps were carried out according to the reagent instructions. The dilution concentrations were 1:150 and 1:200, respectively. TNFSF14 was positive in the cytoplasm, DUSP9 was positive in the nucleus of tumor cells, and brown-yellow staining was considered positive.

### Immunotherapy response validation of DUSP9 and TNFSF14

In the absence of a STS immunotherapy cohort, we collected Liu et al.’s cohort (23) (121 melanoma patients) to validate the immune response of two hub genes. Before further analysis, the mRNA data was converted into “FPKM” form. CR/PR (complete response or partial response) represents immunotherapy response, and SD/PD (stable disease or progressive disease) represented no response to immunotherapy.

### Cell culture and reagents

In this experiment, the human liposarcoma cell line SW872 (CL-0685B) and the human fibrosarcoma cell line HT1080 (CL-0117) were purchased from Procell Biotech (Wuhan, China), and passed STR identification and mycoplasma contamination testing. In this study, SW872 and HT1080 were cultured in DMEM (Gibco), which was supplemented with 10% FBS (Gibco) and 1% streptomycin/penicillin (Procell Biotech). To overexpress TNFSF14, TNFSF14 were cloned into a pcDNA3.1 vector, then transiently transfected into SW872 and HT1080 cells for 24 h. Lipofectamine 3000 (Invitrogen, USA) was used for transfections according to the protocol.

### Cell proliferation, migration, and invasion assays

We evaluated the growth curve of Sarcoma cells using CCK-8 (Dojindo, Japan) following the manufacturer’s instructions. For the cell invasion assay, we applied chambers (8 μm pore size, CORNING) with Matrigel (R&D Systems, USA). We suspended 5×104 cells in 200 serum-free μL medium and added them to the upper chambers. The bottom chambers contained DMEM medium (600 μL) that had 10% FBS as a chemoattractant. After incubating at 37°C for 24 hours, we stained the cells that migrated to the other side of the membrane with 0.1% crystal violet solution; we calculated the results using a microscope (Olympus, Japan).

### Western blotting

For the Western blot assay, we blocked the membranes with 5% skim milk in TBST and incubated them with the indicated primary antibodies overnight at 4°C, followed by the secondary horseradish peroxidase (HRP)-conjugated secondary antibodies. We obtained the following antibodies from Proteintech: antibodies against TNFSF14 (PA00110) and antibodies against GAPDH. The Chemiluminescent Imaging System (Tanon 5200, China) was applied to visualize protein expression signals; the expression of GAPDH served as internal control.

### Flow cytometry

The fresh tissues collected from leiomyosarcoma (LMS) patients and dedifferentiated liposarcoma (DDL) patients were cleaned with PBS and cut into pieces. We further dissociated the shredded tissue into a single-cell suspension, and the Tumor Dissociation Kit (miltenyi, Germany) was used for dissociation according to the protocol. We collected single-cell suspension and washed with PBS before labeling with fluorescent antibodies CD3, CD8, CD11c, and CD19 (Biolegend). We incubated it at 4° C for 30 minutes, washed it three times with PBS, and then resuspended the cells for flow cytometry detection.

### Statistical analysis

All statistical analyses and pictures were conducted under R (version 4.1) and GraphPad. To evaluate continuous variables, we utilized the Wilcoxon test (Mann-Whitney test), while categorical data was analyzed using Fisher’s exact test or chi-square test. The Kaplan-Meier, univariable, and multivariable Cox analysis with log-rank test were used to determine the survival difference. Log-rank test was used for univariable and multivariable Cox proportional hazards analyses. The false discovery rate (FDR), also called adjusted *P* value was used to select the DEGs between TLS-H and TLS-L patients. *P*<0.05 is the statistically significant difference threshold.

## Results

### Four TLS signature establishment and correlation analysis

**Figure 1:**
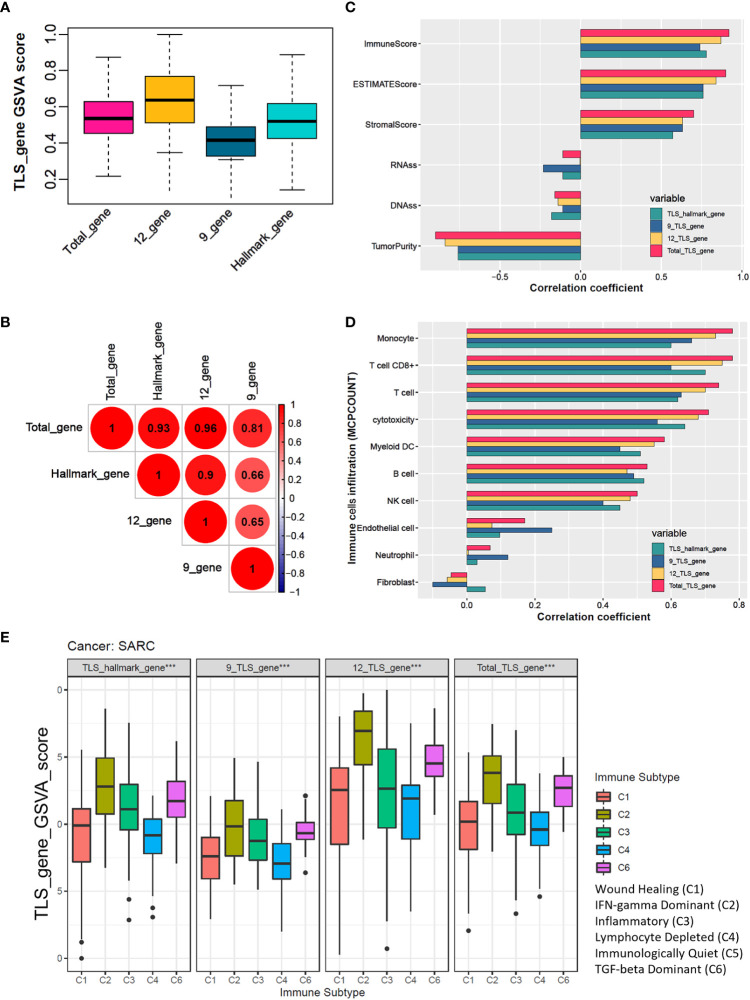
The total TLS gene score is an effective indicator for evaluating the TLS abundance in soft tissue sarcoma samples. **(A)** The TLS relates gene sets enrichment scores among hallmark_genes, 9_TLS_gene, 12_TLS_genes, and total_TLS_gene. **(B)** Correlation coefficients among the above 4 TLS scores with DNAss, RNAss, immune, stromal, estimate scores, and tumor purity. **(C)** Spearman correlation analysis among 4 TLS scores. **(D)** Correlation coefficients among the above 4 TLS scores with 10 immune cells were estimated via the “MCPcounter” algorithm. **(E)** Comparison of 4 TLS scores between different immune subtypes, the C5 immune subtype was not identified in the TCGA sarcoma cohort, ***: P<0.001.

The analysis workflow is shown in **Supplementary Figure 1**. We estimated four TLS signatures based on published literature via the GSVA algorithm. The box plot showed that the total-gene TLS score abundance is in the middle level among the scores of hallmark-gene, 12-gene, and 9-gene (**Figure 1A**). Further correlation analysis showed that the total-gene TLS score has a strong correlation with the score of hallmark-gene, 12-genes, and 9-gene, with correlation coefficients of 0.93, 0.96, and 0.81, respectively (**Figure 1B**). We calculated the immune, stromal score and tumor purity of each sample via the “ESTIMATE” algorithm, and further analyzed their correlation with four TLS scores. The total-gene TLS score has the highest correlation with the immune score, ESTIMATE score, and stromal score, with correlation coefficients of 0.92, 0.9, and 0.7 (**Figure 1C** and **Supplementary Figure 2**, all *P*<0.05), respectively. Otherwise, the total-gene TLS score has a higher negative correlation with tumor purity, RNAss, and DANss, with correlation coefficients of -0.9, -0.11, and -0.16, respectively (**Figure 1C** and **Supplementary Figure 2**, *P*<0.001, *P*=0.011 and *P*= 0.071). We further calculated the abundance of immune cells with MCPCOUNT algorithm, and the correlation coefficients between TLS-Total and T cells, CD8+T cells, cytotoxicity score, NK cells, B cells, monocytes, endothelial cells, and myeloid dendritic cells were 0.74, 0.78, 0.71, 0.5, 0.53, 0.78, 0.58, 0.17 (**Figure 1D** and **Supplementary Figure 3**; all *P*<0.05, except for neutrophils, endothelial cells, and fibroblasts). Similar results were also found in the TIMER2.0 database, which concluded 6 immune cells (**Supplementary Figure 4**). The abundance of immune cells in the total-gene TLS score was superior to the other three TLS scores, which indicated that the total-gene TLS score reflected the level of TLS infiltration in STS more accurately. We divided patients with soft tissue sarcoma into 5 subtypes of tumors based on previous studies, among which the C5 subtype was not identified in the TCGA-SARC cohort. We compared the TLS scores of 5 different immune subtypes (**Figure 1E**, all P<0.05), and the results showed that the C2 (IFN-γ-dominant) subtype had the highest TLS score, followed by C6 (TGF-β-dominant subtypes), C3 (inflammatory), C1 (wound healing), and C4 (lymphocyte-depleted) subtypes. Which indicated that the TLS score was associated with immune activation and stimulation ability.

### Clinical characteristics and differential gene enrichment analysis between high and low TLS subtypes

**Figure 2:**
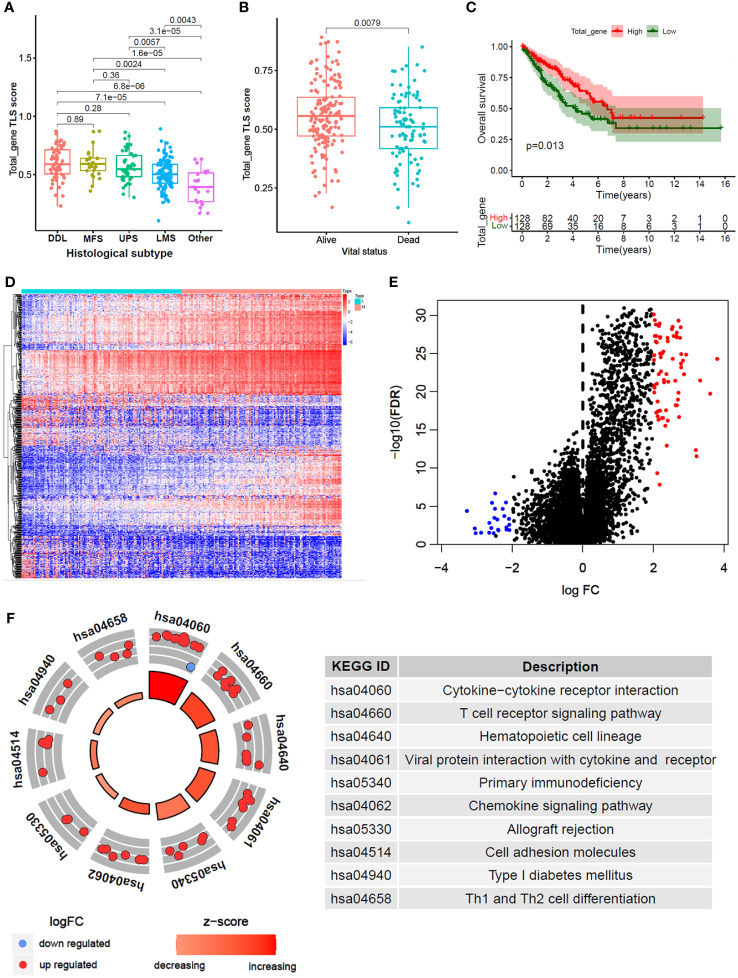
Differential gene analysis and functional annotation between high and low total_gene TLS score. **(A, B)** Comparison of total_gene TLS score between different histological subtypes **(B)** and different vital status **(B)**. **(C)** Kaplan-Meier analysis between high and low total_gene TLS score subtypes. **(D)** Expression heatmap and volcano plot **(E)** of different expression genes between high and low total_gene TLS score subtypes (FDR<0.05 and |logFC|>2). **(F)** KEGG pathways enrichment of selected different expression genes.

A total of 256 STS samples were evenly divided into TLS-H and TLS-L two subgroups based on total-gene TLS score (**Table 1**). The chi-square test showed that (**Figures 2A, B**), the TLS-H group was older (*P*=0.001), had more male patients (*P*<0.001), and had more undifferentiated liposarcoma (DDL), while the proportion of leiomyosarcomas (LMS) was lower (*P*<0.001), and the mortality rate of patients was lower (*P*=0.029). In the comparison of total-gene TLS scores among different histological subtypes (**Figure 2A**), the boxplot showed that DDL and Myxofibrosarcoma (MFS) have higher TLS scores, followed by undifferentiated pleomorphic sarcoma (UPS), LMS, and other subtypes. The STS patients under alive vital status have higher total-gene TLS scores than those of dead-status patients (**Figure 2B**, *P*=0.008). In addition, the TLS-H patients had a better overall survival benefit (**Figure 2C**, *P*=0.013).

Furthermore, 96 DEGs were selected between TLS-H and TLS-L subgroups (**Figures 2D, E**; |log2FC|>2.0 and FDR<0.05). and the DEGs were enriched in immune-related pathways (**Figure 2F**), such as **“**Cytokine-cytokine receptor interaction”, “T cell receptor signaling pathway” and “Th1 and Th2 cell differentiation”. In addition, we explored the clinical features (age and gender) and progression free survival (PFS) analysis of total TLS score in our new manuscript (**Supplementary Figures 5A–C**). The results showed that the elder and male lead to high TLS score, which might due to chronic inflammatory stimulation while the underlying mechanism was not yet clear. Furthermore, we compared the fraction of immune cells infiltration between TLS-high and TLS-low subtypes (**Supplementary Figures 5D, E**). The results showed that TLS-high subtype was enriched in T cells, B cells macrophage cells, while low enriched in fibroblast.

### Construction and validation of TLS-related genes prognosis risk score

**Figure 3:**
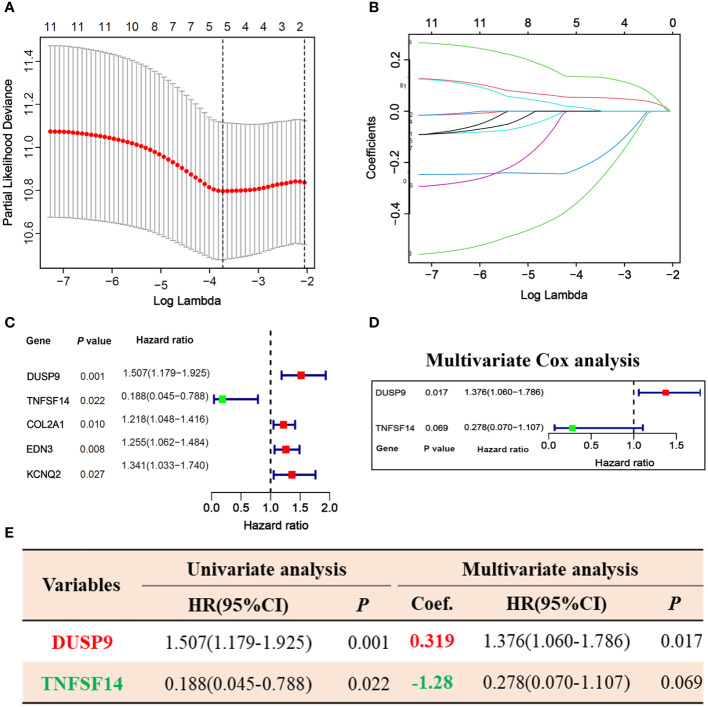
Prognosis-related hub genes selection via univariate, LASSO, and multivariate Cox regression in the training cohort. **(A, B)** 10-fold cross-validation plot **(A)** and TLS-related gene selection **(B)** by LASSO regression. **(C)** Forest plot of 5 LASSO selected genes in univariate Cox analysis. **(D)** Forest plot of 2 hub genes selected by multivariate Cox analysis. **(E)** Univariate and multivariate Cox regression analysis of 2 hub genes.

**Figure 4:**
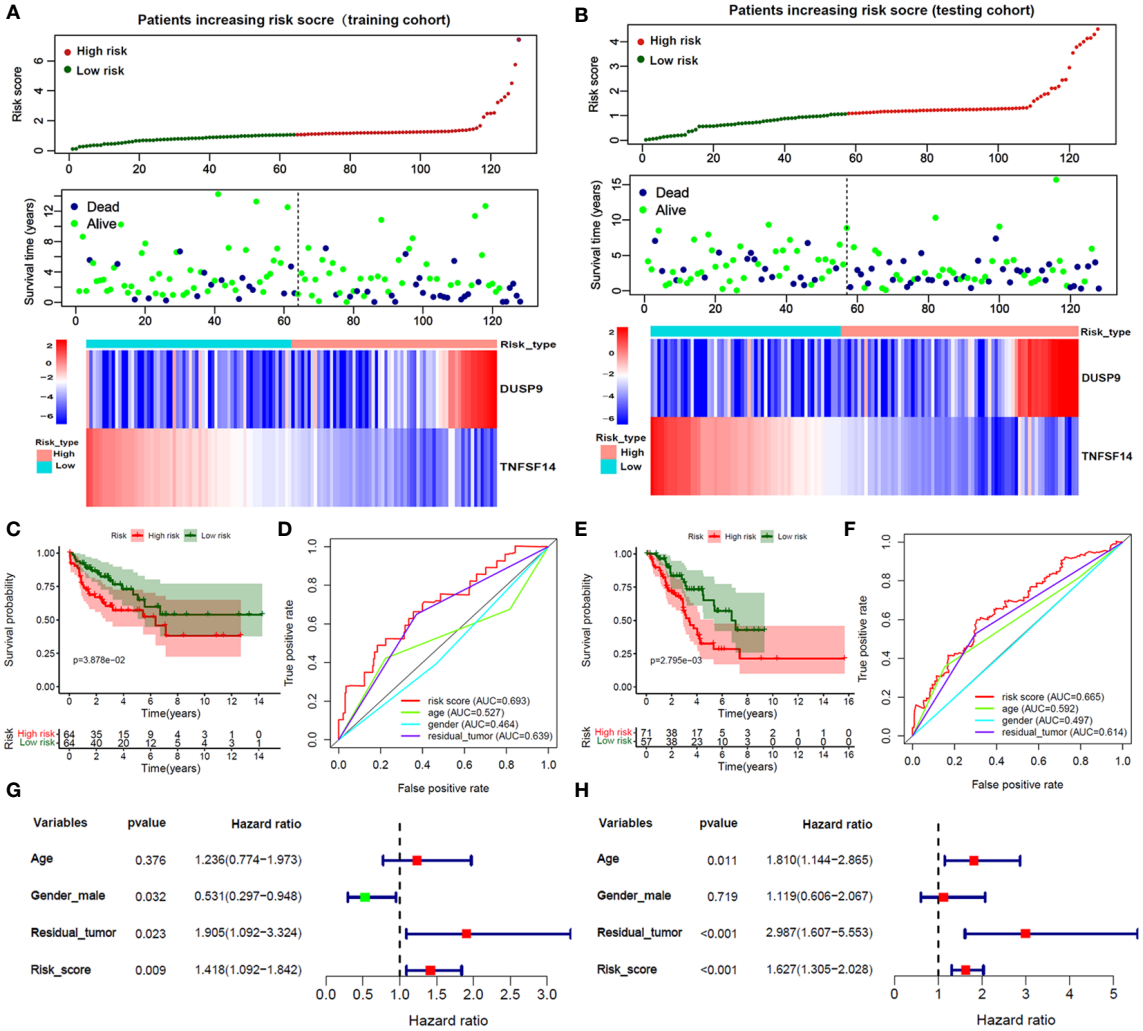
Validation of Risk score in training and testing cohorts. **(A, B)** The survival time, vital status, and 2 hub genes expression of patients ranked by Risk score in the training and testing cohorts. **(C, E)** The Kaplan-Meier analysis between high and low-risk score subgroups in the training and testing cohorts. **(D, F)** The ROC curves of the risk score and clinical features in predicting 1-year OS rate in the training and testing cohorts. **(G, H)** Forest plot of risk score and clinical features by multivariate Cox analysis in the training and testing cohorts.

256 STS samples were randomly divided into training (n=128) and testing (n=128) cohorts. There is no significant difference in clinical characteristics such as age, gender, TLS subtype, histological, vital status, and residual tumor (**Supplementary Table 1**). We applied univariate and LASSO-Cox regression analysis (**Figures 3A, B**), and selected 5 prognosis significant genes (**Figure 3C**; DUSP9, TNFSF14, COL2A1, EDN3, and KCNQ2). Finally, we identified two prognostic TLS-related genes (DUSP9 and TNFSF14) by multivariate COX analysis (**Figures 3D, E**). In the training cohort, the patients with increasing risk scores have a high fraction of dead vital status, higher DUSP9 expression, and lower TNFSF14 expression (**Figure 4A**). Similar results were found in the testing cohort (**Figure 4B**). As expected, the patients with lower risk scores have better overall survival benefits in both training and testing cohorts (**Figures 4C, E**).

### Evaluation of prognostic value of score and clinical features

We applied the ROC curve to evaluate the accuracy of predicting 1-year OS in patients. In the training cohort (**Figure 4D**), the risk core had the highest AUC value, followed by the residual tutor (0.639) age (0.527), and gender (0.464). And the testing cohort (**Figure 4F**) had similar results that the AUC value of the risks core was 0.665, followed by the residual tutor (0.614) age (0.592), and gender (0.497). We further adjusted the risk score with clinical information such as age, gender, and residual tumor. The forest plot showed that the risk score in training and testing sets was an independent risk factor for patient OS (**Figures 4G, H**).

### Tumor microenvironment analysis of STS in single-cell dataset

**Figure 5:**
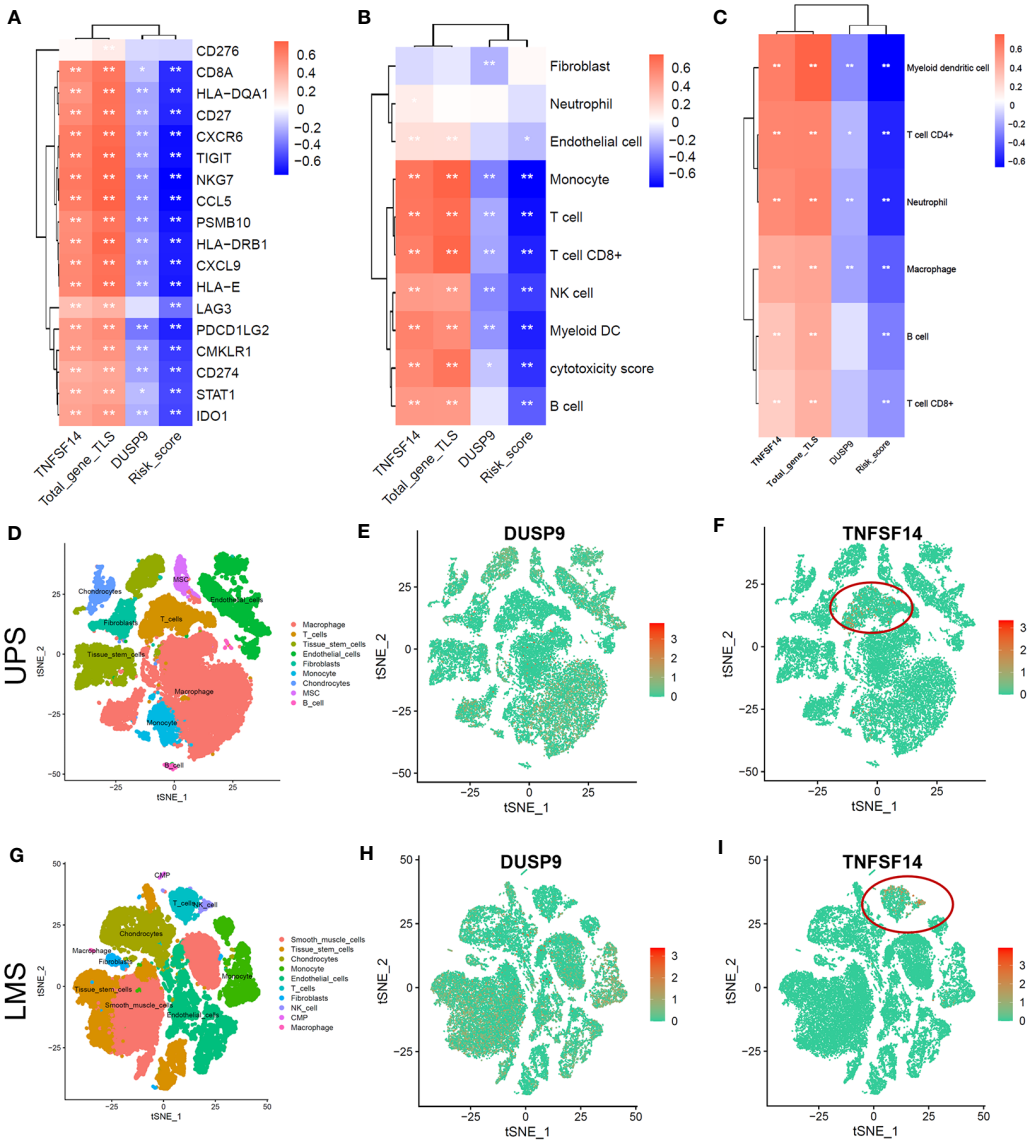
Tumor microenvironment analysis of soft tissue sarcoma in TCGA and single-cell dataset. **(A-C)** Correlation heatmap between total-gene TLS score, Risk score, DUSP, and TNKSF14 with 18 immune-related genes **(A)**, MCPcounter immune cells **(B)**, and TIMER2.0 immune cells **(C)**. **(D-F)** The tSNE plot of Tumor microenvironment cell-clusters **(D)**, DUSP9 **(E)**, and TNFSF14 expression **(F)** in undifferentiated pleomorphic sarcoma (UPS) patients. **(G-I)** The tSNE plot of Tumor microenvironment cell-clusters **(G)**, DUSP9 **(H)**, and TNFSF14 expression **(I)** in leiomyosarcoma (LMS) patients.

We explored the correlation between 18 immune-related genes and TNFSD14, DUSP9, total-gene TLS score, and risk score. The correlation heatmap showed that TNFSF14 and total-gene TLS score were significantly positively correlated with 18 immune-related genes, while DUSP and Risk scores were significantly negatively correlated (**Figure 5A**, *P*<0.05). In addition, TNFSF14 and total-gene TLS score were significantly positively correlated with T cell, B cell, CD8+ T cell, and dendritic cell, while DUSP and Risk score were significantly negatively correlated (**Figures 5B, C**, *P*<0.05). We further explored the immune microenvironment of STS in the single-cell dataset (GSE212527 cohort). The tumor microenvironment in leiomyosarcoma (LMS) is mainly composed of 9 clusters, including T cells, B cells, macrophages, fibroblasts, etc. (**Figure 5D**); and the undifferentiated pleomorphic sarcoma (UPS) is mainly composed of 10 clusters, including T cells, B cells, monocytes, NK cells, etc. (**Figure 5G**). The expression of DUSP was enriched in Macrophage and endothelial cell clusters in both UPS and LMS samples (**Figures 5E, H**). Otherwise, the expression of TNFSF14 was enriched in only T cell clusters in both UPS and LMS samples (**Figures 5F, I**). These indicated that TNFSF14 might assist T cells and exert anti-tumor immune regulatory effects.

### Expression analysis of DUSP9 and TNFSF14 in different STS subtypes and immunotherapy responses

**Figure 6:**
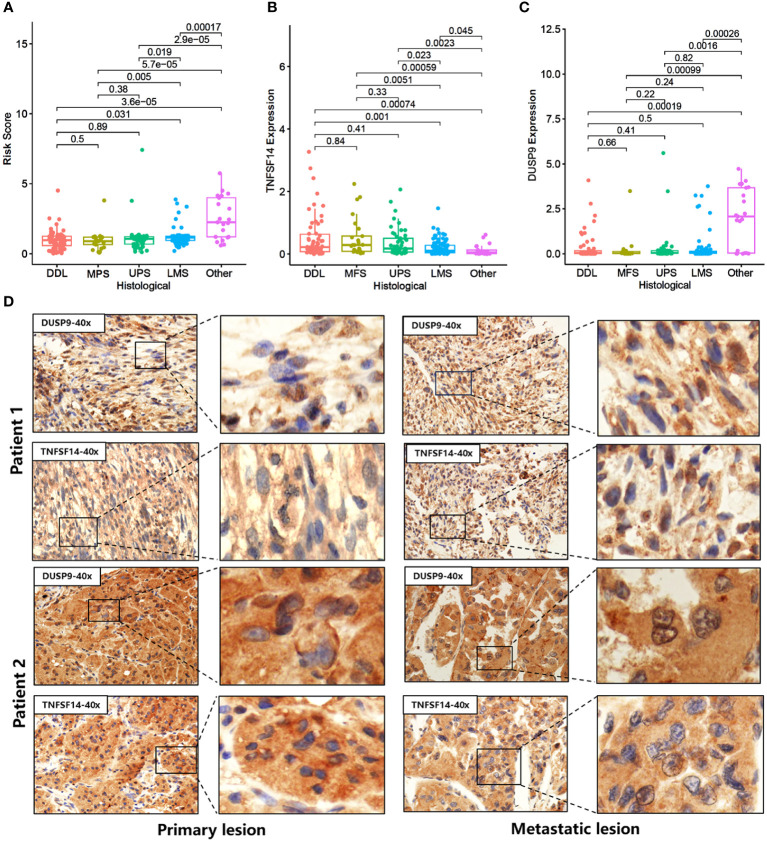
Expression of DUSP9 and TNFSF14 in STS patients receiving immunotherapy. **(A-C)** Comparison of Risk score **(A)**, TNFSF14 **(B)**, and DUSP9 **(C)** among different histological subtypes. **(D)** Immunohistochemical staining of DUSP9 and TNFSF14 in the primary and metastatic lesions of two soft tissue sarcoma patients under immunotherapy.

We compared the differences in Risk score, TNFSF14, and DUSP9 among different STS subtypes. The results showed that TNFSF14 was highly expressed in DDL, MFS, and UPS, and downregulated in LMS and other subtypes, while the Risk score and DUSP9 were the opposite (**Figures 6A–C**). To further analyze the immunotherapy response of DUSP9 and TNFSF14, we collected primary site tumors and metastatic tissue from two locally advanced alveolar soft part sarcoma (ASPS) patients who received immunotherapy combined with anti-angiogenic drugs, matched with clinical features in our center. The immunohistochemical results showed that TNFSF14 was positively expressed in the cytoplasm, DUSP9 was positively expressed in the nucleus of tumor cells, and brown-yellow staining was considered positive (**Figure 6D**). As expected, the staining of primary tumor tissue and lung metastases showed that TNFSF14 was positively expressed as a prognostic protective factor, and DUSP9 was negatively expressed as a prognostic risk factor. In addition, we further explored the immune response of two hub genes in public cohort deficient in STS immunotherapy. The results showed that compared with SD/PD subtypes, patients with CR/PR subtype had higher expression of TNFSF14 and lower expression of DUSP9 (**Supplementary Figures 6A, D**). Patients with higher expression of TNFSF14 (**Supplementary Figures 6B, C**) or lower expression of DUSP9 (**Supplementary Figures 6E, F**) had better overall survival (OS) and progression -free survival (PFS). This indicated that high TNFSF14 and low DUSP9 might be potential biomarker for better immunotherapy response.

### Flow cytometry analysis of immune cell distribution in soft tissue sarcoma

**Figure 7:**
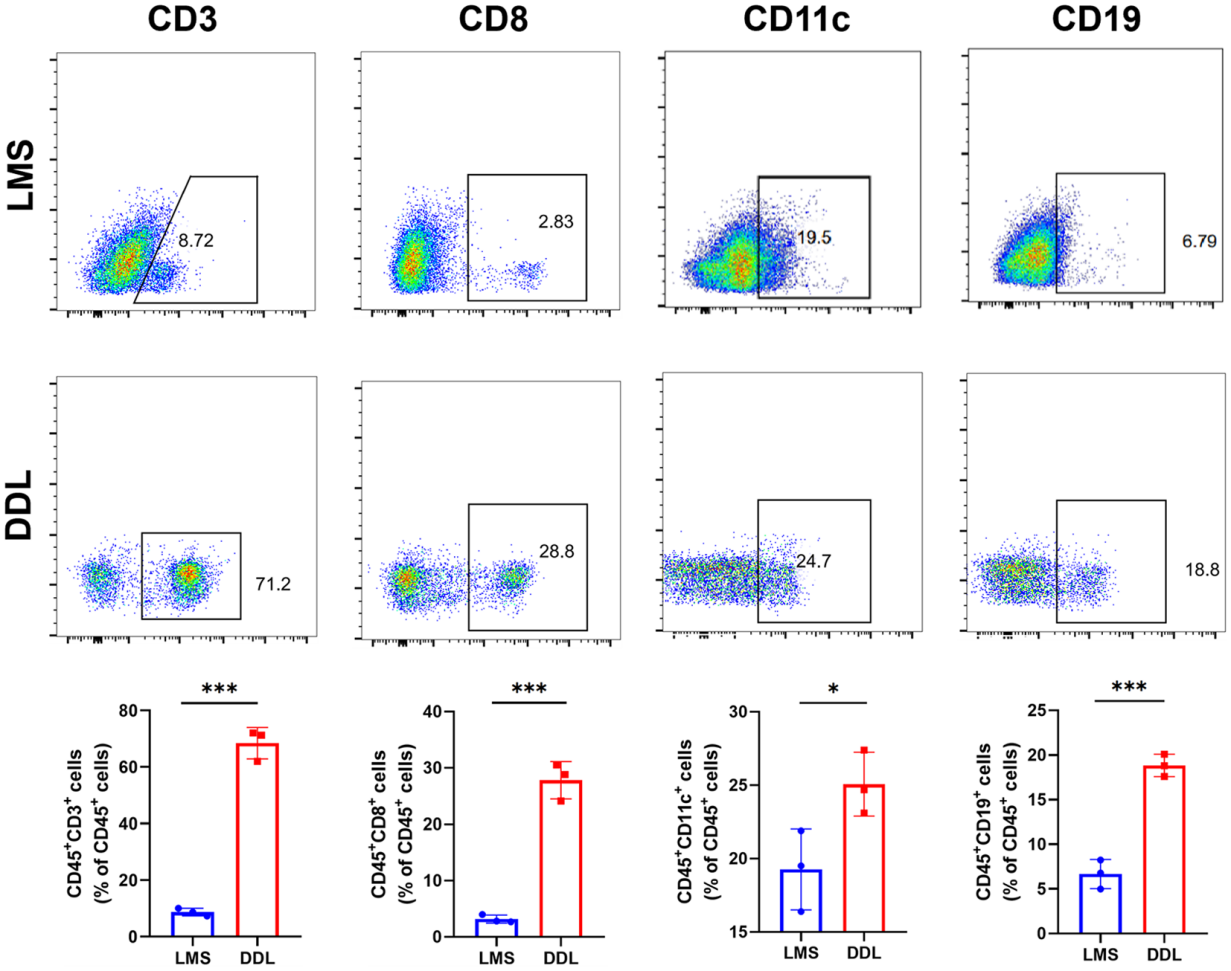
Analysis of the abundance of immune cell infiltration in leiomyosarcoma (LMS) and dedifferentiated liposarcoma (DDL) patients. Flow cytometry results showed the abundance of each 4 types of infiltrating cells in LMS and DDL. The results of statistical analysis showed the proportion of cell infiltration. *P ≤ 0.05, ***P ≤ 0.001.

To clarify the abundance of immune cell infiltration in the tumor microenvironment of soft tissue sarcoma, we applied flow cytometry to detect different proportions of immune cells in DDL and LMS sarcoma. The results showed that the infiltration of CD3, CD8, CD19, and CD11c positive immune cells significantly increased in DDL, indicating that the infiltration levels of T cells, B cells, and DC in their microenvironment were significantly higher than those in LMS (**Figure 7**). These results demonstrated that the tumor microenvironment in DDL was dominated by infiltration of activated immune cell types.

### TNFSF14 overexpression suppresses sarcoma cell proliferation and migration

**Figure 8:**
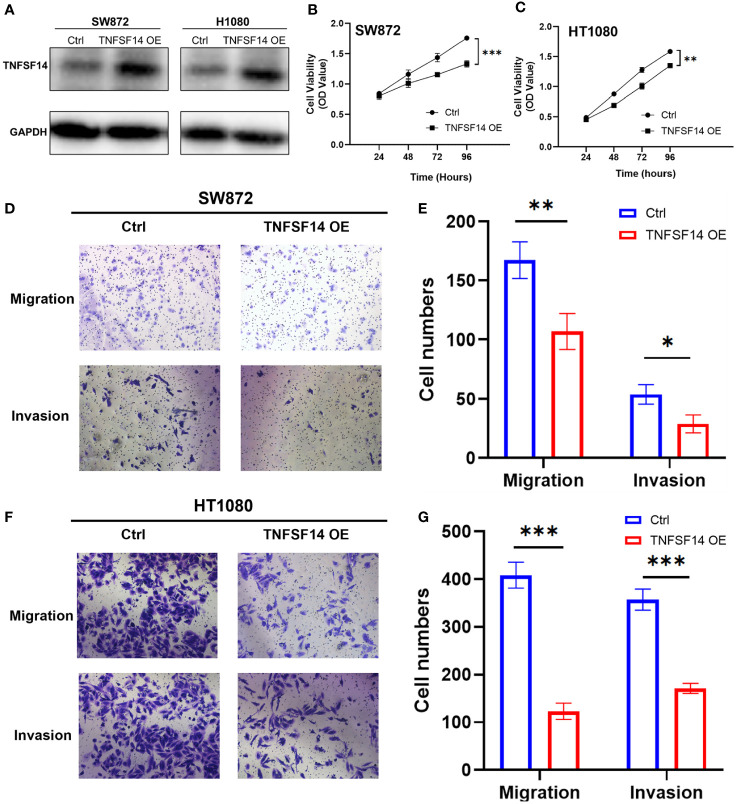
Validation of the function of TNFSF14 by *in vitro* experiments. **(A)** TNFSF14 overexpressed was confirmed by western blot assays in SW872 and HT1080 cells. **(B, C)** CCK-8 assays showed reduced cell proliferation in TNFSF14 overexpressed cells. **(D–G)** Cell migration and invasion of sarcoma cells were determined by Transwell assays. *P ≤ 0.05, **P ≤ 0.01, ***P ≤ 0.001, ns, no significance.

To determine the functional role of TNFSF14 in sarcoma, we overexpress TNFSF14 in SW872 and HT1080 cells with the overexpression plasmid vector. Overexpression of TNFSF14 was validated in western blots (**Figure 8A**). The results clearly showed that overexpression of TNFSF14 significantly inhibited the proliferation of both SW872 and HT1080 cells (**Figures 8B, C**). Transwell assay showed that overexpressing TNFSF14 significantly inhibited cell migration and invasion in SW872 and HT1080 cells (**Figures 8D–G**).

## Discussion

Tertiary lymphoid structures (TLS) are an important component of the tumor immune microenvironment and play an important coordinating role in the coordinated immune response of tumors (11). TLS is mainly composed of B cells and T cells, and mature TLS also includes follicular dendritic cells and germinal centers (24, 25). Based on transcriptome analysis, several gene features have been used to detect TLS, including 12 chemokine features, TFH cell features, and TH1 and B cell features (26). To ensure the successful detection of all TLS, we established a 28-gene signature to estimate the heterogeneity of TLS in soft tissue sarcoma. In this study, we established four TLS-related scores that can reflect the relative abundance of TLS in the tumor microenvironment based on previous literature. We identified that the total TLS gene score is highly correlated with immune cell infiltration, and all three TLS scores also exhibit high heterogeneity, enabling a more accurate assessment of tumor TLS abundance.

The tertiary lymphatic structure plays an important role in predicting tumor prognosis. We analyze the clinical features relationship between high and low TLS subtypes. The DDL histological subtype has the highest total-gene TLS score, and patients with higher TLS scores have a better overall survival benefit. We further identified two hub genes, DUSP and TNFSF14, which were significantly correlated with TLS and prognosis. The bispecific phosphatase (DUSP) family plays a crucial role in maintaining cell homeostasis and apoptosis (27). DUSP9 reduces liver ischemia/reperfusion injury by inhibiting mitogen-activated protein kinase and IKK in a kinase 1-dependent manner through cell apoptosis signaling regulation (28). DUSP9 functions differently in different types of cancer and has not been reported in soft tissue sarcoma. The decrease in pERK1/2 levels mediated by DUSP9 affects the stem cell-like characteristics of triple-negative breast tumors, promoting tumor cell growth (29). Otherwise, compared with normal tissues surrounding colorectal cancer, DUSP9 is significantly downregulated in tumor tissues (30). The high methylation status of CpG islands in the DUSP9 promoter may lead to the downregulation of DUSP9 in (colorectal cancer, CRC), while upregulation of DUSP9 can inhibit the proliferation, migration, invasion, and epithelial-mesenchymal transition of CRC cells (30). This study shows a significant negative correlation between DUSP and immune cell infiltration and suggests poor prognosis and immune therapy response in patients. In addition, studies have shown that overexpression of DUSP9 can enhance the proliferation of liver cancer cells *in vitro*, and high expression of DUSP9 is associated with shorter disease-free survival, suggesting that DUSP9 may also be a new prognostic indicator and therapeutic target for liver cancer (31). Further studies are required to determine the specific mechanisms. TLSs have been reported to be associated with increased infiltration of B and T cells (32). We found that the TLS hub gene, TNFSF14, was enriched in a T-cell cluster based on single-cell sequencing analysis. Tumor necrosis factor superfamily protein 14 (TNFSF14), also known as LIGHT, is an important regulatory factor in immune and fibrotic diseases (33). TNFSF14 and T cell co-stimulatory molecules can activate NK cells, but cannot directly kill tumor cells. Instead, they utilize IFN-γ promoting the initiation of tumor-specific CD8+T cells (34). Zhang N. et al. found that the expression of TNFSF14 is highly correlated with TLS-hallmarked genes, which can promote CAR-T cell transport and cytotoxicity by reversing the immunosuppressive tumor microenvironment (35). In glioblastoma, TNFSF14 is involved in T cell activity and inflammatory processes, which can indicate patient prognosis and immunotherapy efficacy. In addition, overexpression of TNFSF14 can promote the formation of T-cell-rich TLS and effective anti-tumor T cell response, prolonging the survival of glioma cells, indicating that TNFSF14 may be a biomarker for predicting immunotherapy response (36). Our cell experiments also showed that the proliferation, invasion, and migration of STS cell lines overexpressing TNFSF14 were significantly inhibited.

Our research suggests that the expression of DUSP9 and TNFSF14 may influence the functional dynamics of tertiary lymphoid structure, which plays an important role in regulating local immune responses and promoting immune cell infiltration. These structures serve as hubs for antigen presentation and T cell activation, potentially influencing tumor immunogenicity and overall immune response. Furthermore, the interaction of DUSP9 and TNFSF14 with STS immune microenvironment may be key to tumor escape. DUSP9 and TNFSF14 can affect the recruitment and activation of T, B, dendritic cells, and other immune cells by changing the signaling pathways in the tumor milieu, thus affecting the efficacy of immunotherapeutic agents. The effect of DUSP9 and TNFSF14 on the response to immunotherapy, particularly immune checkpoint inhibitors, warrants further investigation. Further study of the key molecular mechanisms of DUSP9 and TNFSF14 in regulating immune response can provide new prognostic insights and therapeutic targets for STS patients, and better guide personalized immunotherapy strategies for patients with STS.

In this study, we provided insights into the prognostic significance of tertiary lymphoid structure (TLS) scoring in various soft tissue sarcoma (STS) subtypes and their relevance to immunotherapy efficacy. But there are some limitations to our work. The sample size, albeit sufficient for preliminary conclusions, is relatively small and may not represent the full spectrum of STS heterogeneity. There are also potential biases in data sources, including selection bias, which may also affect the analysis results. Moreover, factors such as the complexity of the tumor microenvironment and patient-specific immune status are not fully considered, which also affect the feasibility of TLS scores as biomarkers. Future research should focus on validating the identified STS biomarkers in a larger sample sizes and more diverse patient cohort to confirm their prognostic value. Integrating TLS scores with other emerging biomarkers could provide a more comprehensive prognostic model for patients. Additionally, exploring the mechanistic pathways linking TLS to the immune microenvironment and the response to immunotherapy will further elucidate the potential of TLS as a therapeutic target. Such investigations will pave the way for more personalized and effective treatment strategies to enhance the clinical outcome of STS patients.

## Conclusion

We systematically explored the TLS and related genes from the perspectives of bioinformatics, clinical features, and cytological experiments. The total-gene TLS score could reflect the relative abundance of TLS in the tumor microenvironment, and the Risk score can predict patient prognosis. The TLS-related hub gene TNFSF14 has a positive correlation with immune cell infiltration and immune checkpoint, which Positive correlation with immune cell infiltration and immune checkpoint related molecules, with certain suggestive value for immune therapy response. The total-gene TLS score, Risk score, and TNFSF14 hub gene might be useful biomarkers for predicting the prognosis and immunotherapy efficacy of soft tissue sarcoma.

## Data availability statement

The original contributions presented in the study are included in the article/**Supplementary Material**. Further inquiries can be directed to the corresponding authors.

## Ethics statement

The studies involving humans were approved by Ethics Committee of the First Affiliated Hospital of Air Force Medical University. The studies were conducted in accordance with the local legislation and institutional requirements. The participants provided their written informed consent to participate in this study.

## Author contributions

X-XW: Conceptualization, Data curation, Formal analysis, Investigation, Resources, Software, Validation, Visualization, Writing – original draft, Writing – review & editing. Y-PL: Resources, Validation, Writing – original draft. YL: Resources, Writing – original draft, Data curation, Writing – review & editing. L-HW: Data curation, Resources, Writing – original draft, Investigation, Validation. J-YR: Validation, Writing – original draft, Formal analysis. HJ: Data curation, Writing – review & editing. XW: Writing – review & editing, Resources, Supervision. H-MZ: Writing – review & editing, Conceptualization, Funding acquisition, Validation.
