# Supplementary material for: Identifying specific TLS-associated genes as potential biomarkers for predicting prognosis and evaluating the efficacy of immunotherapy in soft tissue sarcoma
Source: Front Immunol. 2024 Apr 24;15:1372692. doi: 10.3389/fimmu.2024.1372692 (PMC11076739; doi:10.3389/fimmu.2024.1372692)
Supplement: Supplementary file 1 [file DataSheet_1.pdf]

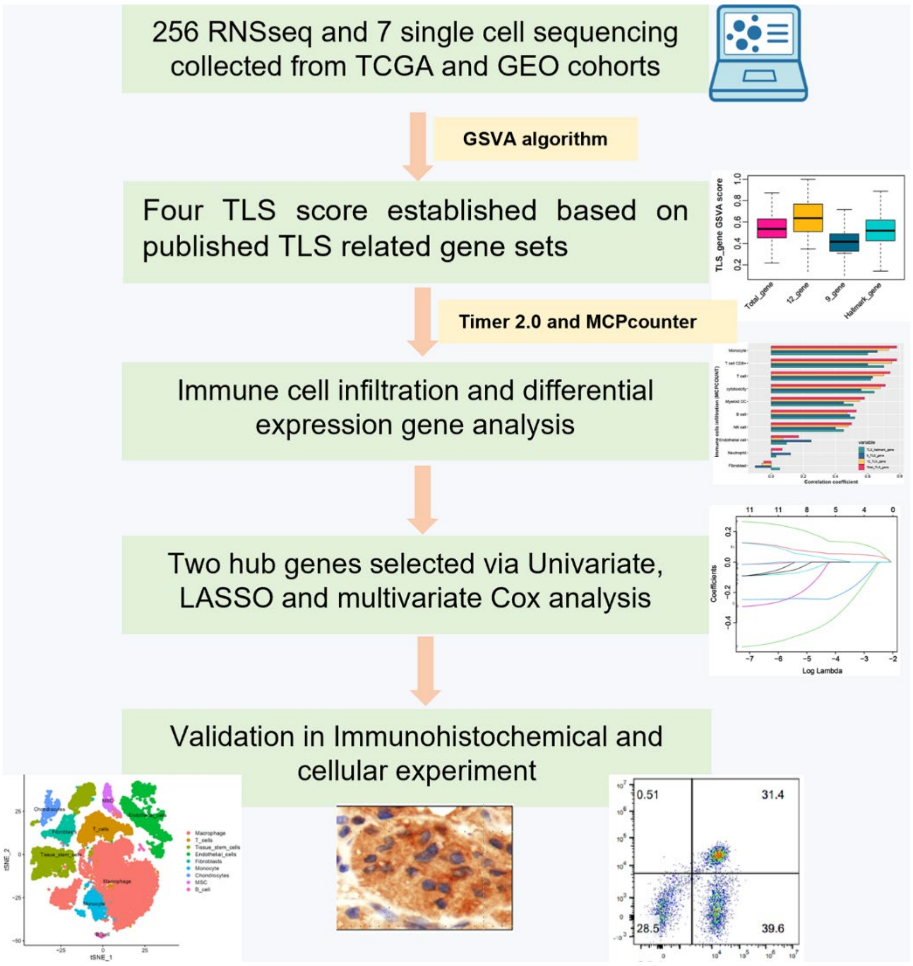

Figure S1. The overview of analysis flowchart.

A total of 256 RNAseq and 7 single cell sequencing collected from TCGA and GEO cohorts. We first applied GSVA algorithm and established four TLS score based on published TLS related gene sets. We estimated the immune cell infiltration via TIMER2.0 and MCPcounter algorithm. In addition, we selected two hub genes, DUSP and TNFSF14, via Univariate, LASSO and multivariate Cox analysis. Finally, we validated our findings in single cell sequencing dataset, clinical immunohistochemical and cellular experiment.

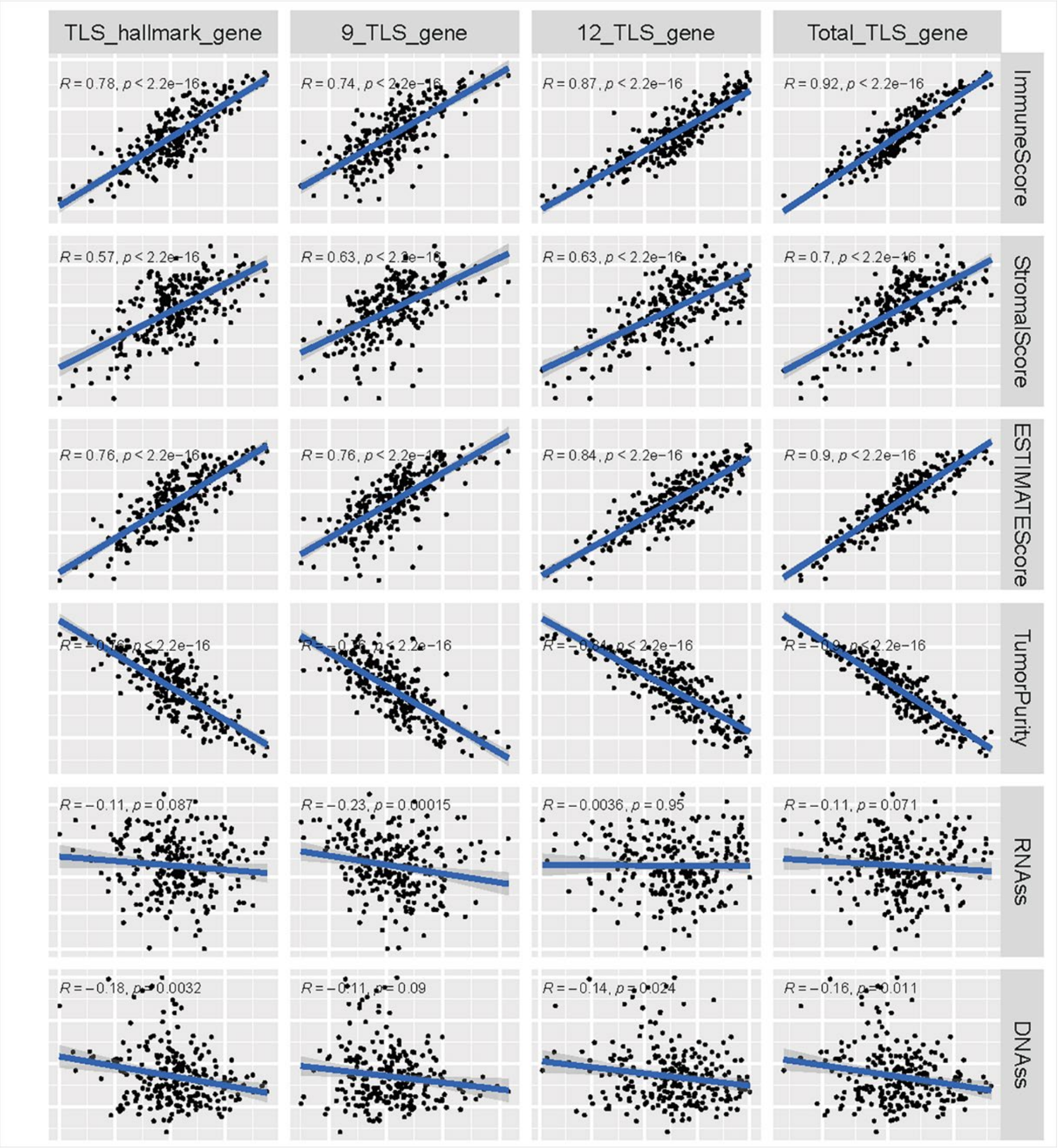

Figure S2. Scatter plot of the correlation between four TLS scores and immune microenvironment.

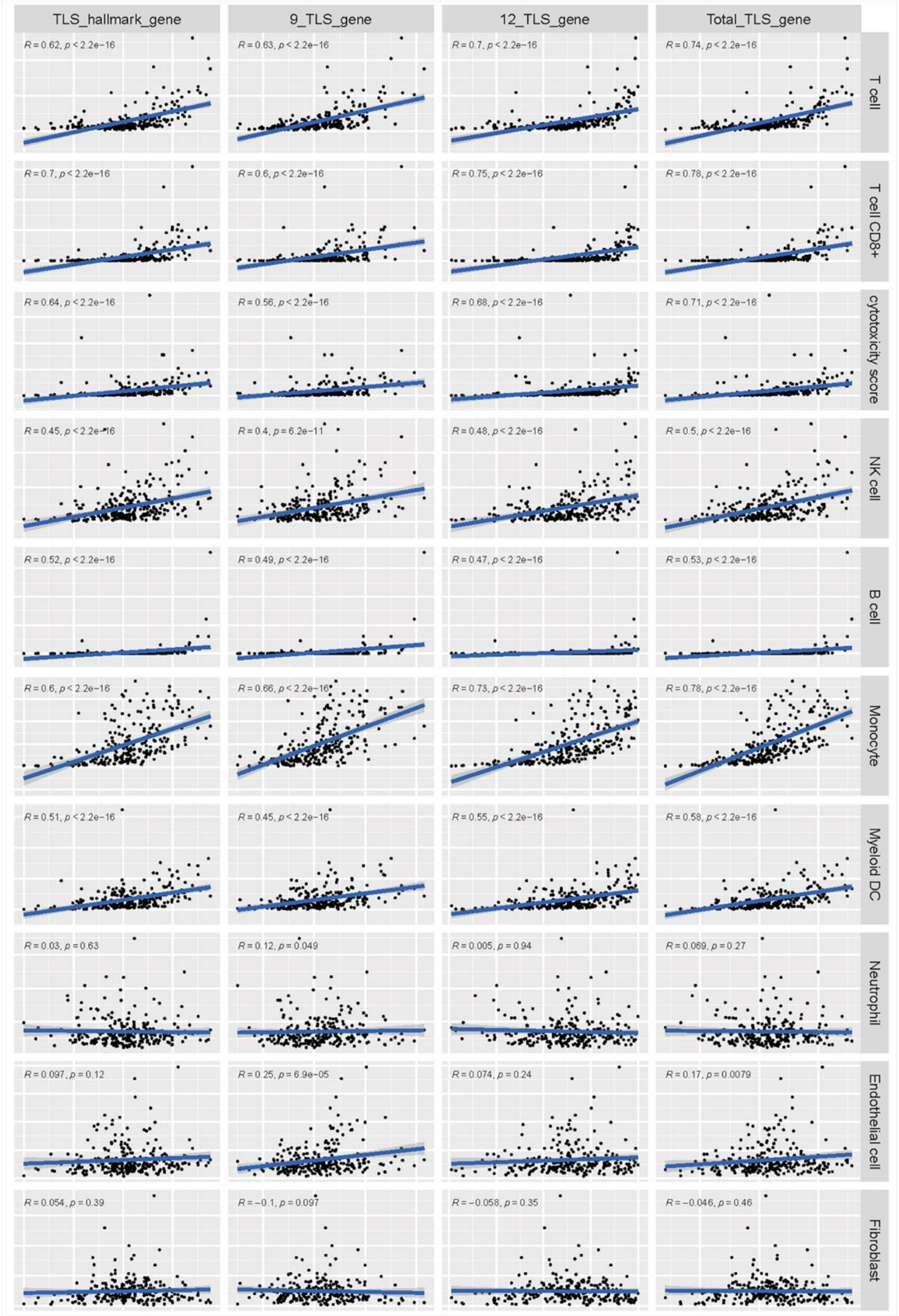

Figure S3. Scatter plot of the correlation between four TLS scores and immune cells infiltration estimated by MCPcounter.

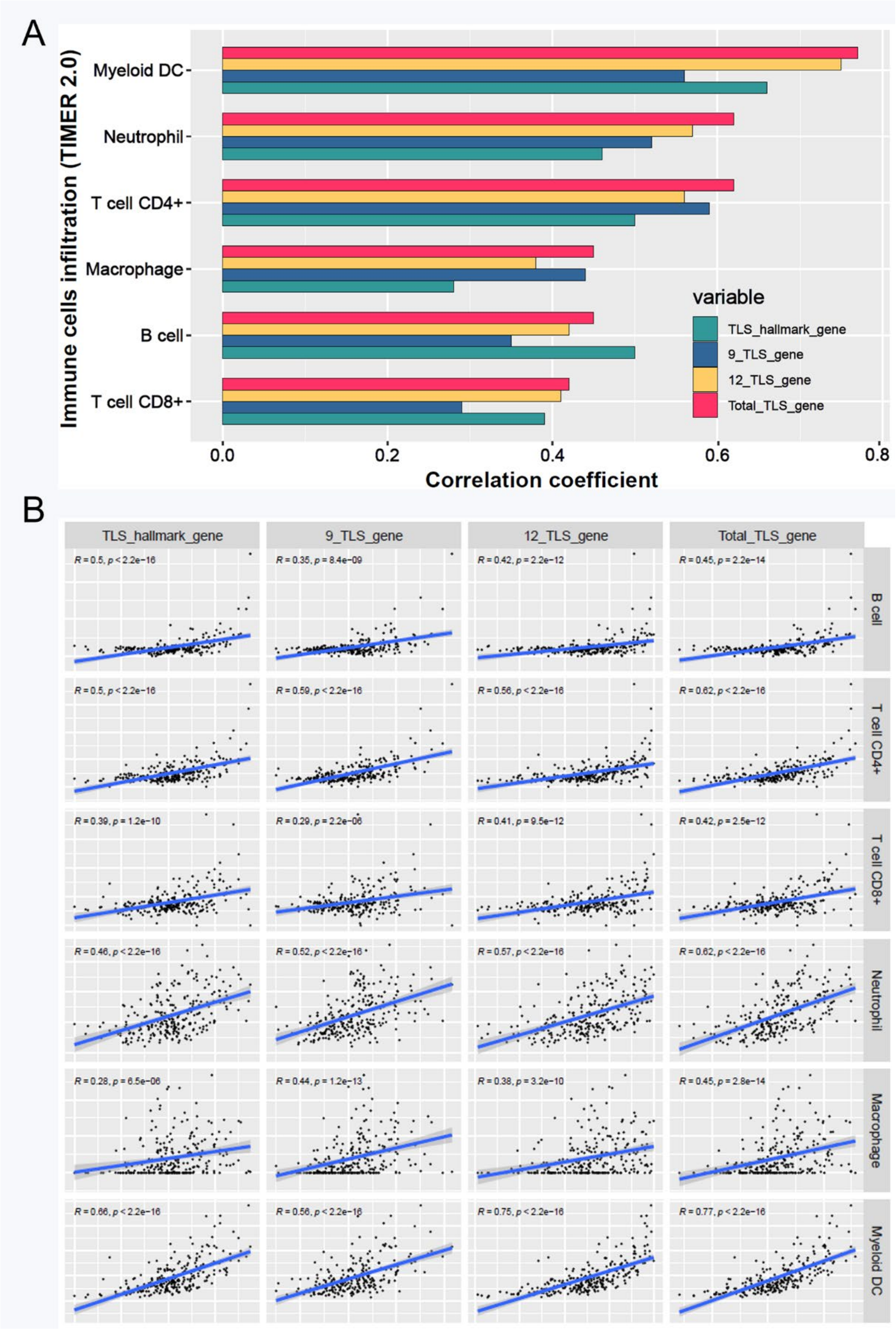

Figure S4. Correlation between four TLS scores and immune cells infiltration estimated by TIMER2.0. (A) Bar-plot and (B) scatter plot of the correlation between four TLS scores and immune cells infiltration estimated by TIMER2.0.

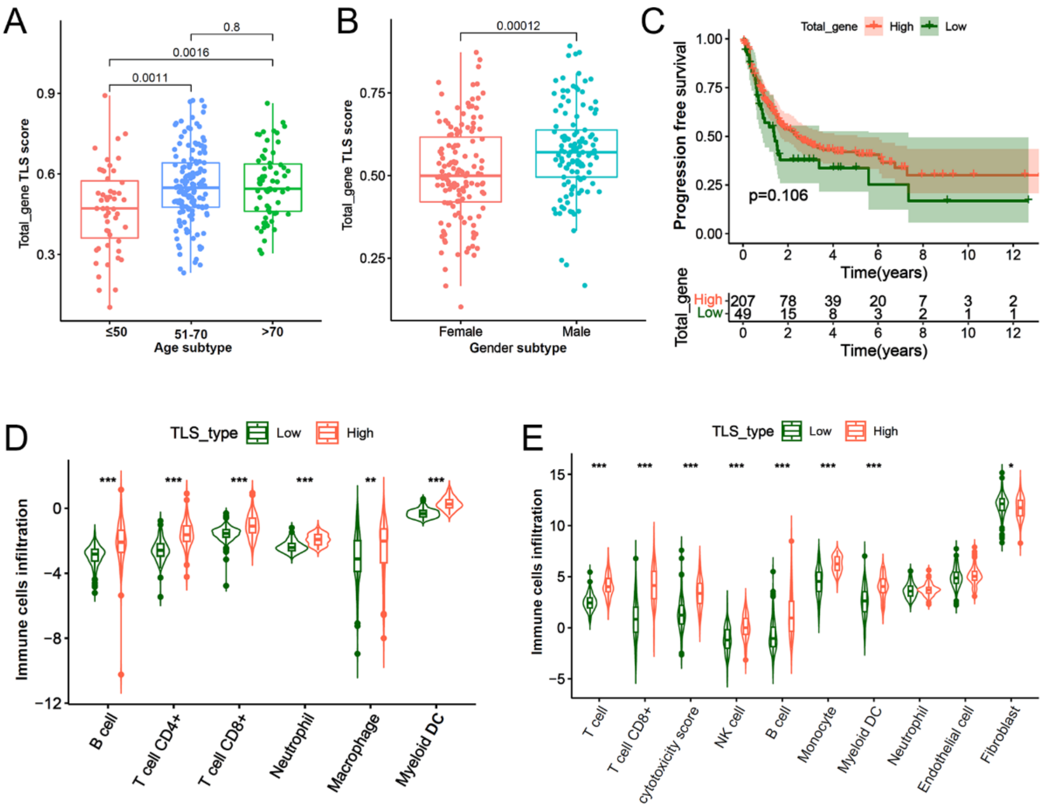

Figure S5. Correlation between four TLS scores and immune cells infiltration estimated by TIMER2.0. (A) Bar-plot and (B) scatter plot of the correlation between four TLS scores and immune cells infiltration estimated by TIMER2.0.

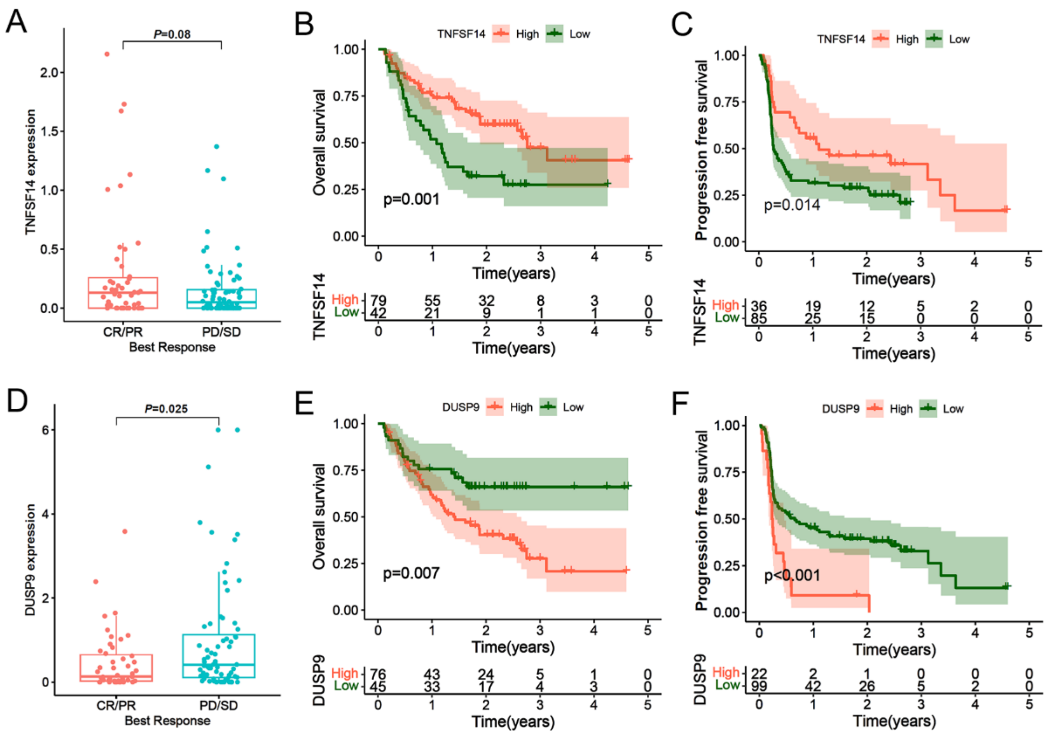

Figure S6. Prognostic relevance of TNFSF14 and DUSP9 in immunotherapy cohort

Table S1. Clinical features of SARC patients in training and testing cohort

Variables	Overall	Training cohort	Testing cohort	*P*
	n=256(%)	n=128 (%)	n=128 (%)	
**Age**				0.058
≤50	51 (19.9)	23 (18.0)	28 (21.9)	
51-70	142 (55.5)	65 (50.8)	77 (60.2)	
>70	63 (24.6)	40 (31.2)	23 (18.0)	
**Gender**				0.316
Female	139 (54.3)	65 (50.8)	74(57.8)	
Male	117 (45.7)	63 (49.2)	54 (42.2)	
**TLS_subtype**				0.169
TLS_L	128 (50.0)	58 (45.3)	70(54.7)	
TLS_H	128 (50.0)	70(54.7)	58 (45.3)	
**Histological**				0.278
DDL	58 (22.7)	27 (21.1)	31 (24.2)	
LMS	103 (40.2)	51 (39.8)	52 (40.6)	
MFS	25 (9.8)	16 (12.5)	9 (7.0)	
UPS	49 (19.1)	27 (21.1)	22 (17.2)	
Other	21 (8.2)	7 (5.5)	14 (10.9)	
**Vital status**				0.247
Alive	158 (61.7)	84 (65.6)	74(57.8)	
Dead	98 (38.3)	44 (34.4)	54 (42.2)	
**Tumor_status**				0.585
Tumor free	125 (48.8)	64 (50.0)	61 (47.7)	
With tumor	122 (47.7)	61 (47.7)	61 (47.7)	
NA	9 (3.5)	3 (2.3)	6 (4.7)	
**Residual_tumor**				0.363
R0	153 (59.8)	76 (59.4)	77 (60.2)	
R1	67 (26.2)	36 (28.1)	31 (24.2)	
R2	9 (3.5)	6 (4.7)	3 (2.3)	
RX	27 (10.5)	10 (7.8)	17 (13.3)	
